# APACHE IV Is Superior to MELD Scoring System in Predicting Prognosis in Patients after Orthotopic Liver Transplantation

**DOI:** 10.1155/2013/809847

**Published:** 2013-11-18

**Authors:** Yueyun Hu, Xianling Zhang, Yuan Liu, Jun Yan, Tiehua Li, Ailing Hu

**Affiliations:** ^1^The Third Affiliated Hospital, Sun Yat-sen University, 600 Tianhe Road, Guangzhou, Guangdong 510630, China; ^2^The Nursing Academy, Sun Yat-sen University, Guangzhou 510080, China

## Abstract

This study aims to compare the efficiency of APACHE IV with that of MELD scoring system for prediction of the risk of mortality risk after orthotopic liver transplantation (OLT). A retrospective cohort study was performed based on a total of 195 patients admitted to the ICU after orthotopic liver transplantation (OLT) between February 2006 and July 2009 in Guangzhou, China. APACHE IV and MELD scoring systems were used to predict the postoperative mortality after OLT. The area under the receiver operating characteristic curve (AUC) and the Hosmer-Lemeshow C statistic were used to assess the discrimination and calibration of APACHE IV and MELD, respectively. Twenty-seven patients died during hospitalization with a mortality rate of 13.8%. The mean scores of APACHE IV and MELD were 42.32 ± 21.95 and 18.09 ± 10.55, respectively, and APACHE IV showed better discrimination than MELD; the areas under the receiver operating characteristic curve for APACHE IV and MELD were 0.937 and 0.694 (*P* < 0.05 for both models), which indicated that the prognostic value of APACHE IV was relatively high. Both models were well-calibrated (The Hosmer-Lemeshow C statistics were 1.568 and 6.818 for APACHE IV and MELD, resp.; *P* > 0.05 for both). The respective Youden indexes of APACHE IV, MELD, and combination of APACHE IV with MELD were 0.763, 0.430, and 0.545. The prognostic value of APACHE IV is high but still underestimates the overall hospital mortality, while the prognostic value of MELD is poor. The function of the APACHE IV is, thus, better than that of the MELD.

## 1. Background 

Liver transplantation has become exclusive and feasible treatment for various end-stage liver diseases, including liver cirrhosis, acute liver failure and tumor [1]. Although liver transplantation is widely conducted, the mortality remains significantly high as much as 5%~8% [2].

As there was no an objective and accurate evaluation tool available for prediction of the outcome for liver transplantation till now [3]. Acute physiology and chronic health evaluation (APACHE) is one of the most widely used and authoritative scoring system for evaluation of the severity and prognosis of critically ill diseases, including liver transplantation, and APACHE IV showed better predicting value against APACHE II and APACHE III [4]. 

The Model for End-Stage Liver Disease (MELD) as another important scoring system for prediction of the mortality of critical ill patients is a survival model. This model has been adopted for donor liver allocation systems in the United States in 2002 [5]. Additionally, MELD has been reported to be used for prediction of the outcome for liver transplantation [6]. 

Therefore, this aims to explore and compare the effects of APACHE IV, MELD and combination APACHE IV with MELD for predicting the mortality risk after orthotopic liver transplantation. 

## 2. Methods and Patients

### 2.1. Patients

A retrospective cohort study was performed. At a liver transplant center in Guangzhou, a total of 195 patients admitted to the ICU after orthotopic liver transplantation (OLT) between February 2006 and July 2009 were included when meeting the inclusion criteria. The inclusion criteria included age > 18 years, patients with OLT for the first time, cadaveric donor (cardiac death) and brain-dead donor liver transplant patients, and patients who had been more than four hours in the ICU after OLT operation. Living donor liver transplantation, multiple organ transplantation, and previous organ transplantation were excluded. 

### 2.2. Data Collection

The data were collected by the researchers independently, and double check was conducted. We recorded the data of the previous day if the data used to calculate the APACHE IV scores was missing, and we recorded the data of the first two days when the data of the previous day was missing.

The APACHE IV scoring system takes age, chronic health conditions, and the acute physiology score (APS) into account. The APS is based upon the worst measurement during the first 24 hrs in the ICU. The Glasgow Coma Scale (GCS) score, whether sedation or paralysis resulted in an inability to assess GCS, and Pao_2_/Fio_2_ were recorded as part of the data collection for APS. All the following were also recorded: ICU admission diagnoses; admission source; length of stay before ICU admission; whether a patient received mechanical ventilation on day 1, had emergency surgery, or was an ICU readmission; and whether a patient with acute myocardial infarction received thrombolytic therapy in the 24 hrs before or after ICU admission. These data were collected over the first 24 hrs of admission to the ICU and were entered into a computer-based APACHE IV calculator. The calculator returns values that include the APACHE score, predicted mortality rate, and predicted ICU length of stay.

The MELD score was determined prior to OLT using the following equation:

(1) MELD=9.57×log⁡e⁡(Creatinine)+3.78×log⁡e⁡(Bilirubin)+11.2×log⁡e⁡(INR)+6.43,

where INR is international normalized ratio and creatinine and bilirubin are expressed in mg/dL. The values lie between 6 and 40, depending on the severity of the clinical conditions.

### 2.3. Statistical Analysis

The outcomes of all patients were presented by frequencies, percentages, mean values, and standard deviations. The correlation between the predicted and the actual ICU LOS was tested using the Spearman test, and the differences were tested using the Wilcoxon test. The discrimination and accuracy of APACHE IV and MELD to predict the early mortality of OLT patients were described by receiver operating characteristic curves (ROC) and the Hosmer-Lemeshow test. The discrimination of a prognostic model is defined as the ability to distinguish between survivors and nonsurvivors. The discrimination of APACHE IV and MELD to predict hospital mortality was analyzed by calculating the area under the receiver operating characteristic curves (AUC). An AUC of >0.9 was considered to be outstanding; 0.7 to 0.9, acceptable; 0.5 to 0.7, poor. The calibration of the model is the degree of agreement between the predicted mortality and actual mortality. The Hosmer-Lemeshow C statistic was used to determine the calibration of the model. A model with good calibration should have a Hosmer-Lemeshow statistic with degrees of freedom, approximately, equal to the number of categories minus 2 as well as a *P*  value > 0.05. Standardized mortality ratios (SMRs) were calculated by dividing the actual rates by the rates predicted by APACHE IV.   The significance level was set at *P* < 0.05. All statistical analyses were performed using SPSS 13.0.

## 3. Results

This retrospective study included 195 adult patients, comprising 171 males and 24 females with an overall mean age of 48.18 ± 11.13 years, who were admitted to the ICU during the immediate OLT postoperative period. Twenty-seven died during hospitalization with a mortality rate of 13.8%; the overall mean APACHE IV and MELD scores were 42.32 ± 21.95, and 18.09 ± 10.55. The mean APACHE IV and MELD scores of nonsurvivors and survivors were 75.26 ± 25.47 versus 35.86 ± 15.58 and 25.70 ± 12.92 versus 16.87 ± 9.61. The mean predicted hospital mortality rates of nonsurvivors and survivors were (12.84 ± 16.18)% and (2.30 ± 3.77)%, respectively (*P* < 0.05). The data was shown in Tables 1, 2, and 3.

**Figure 1:**
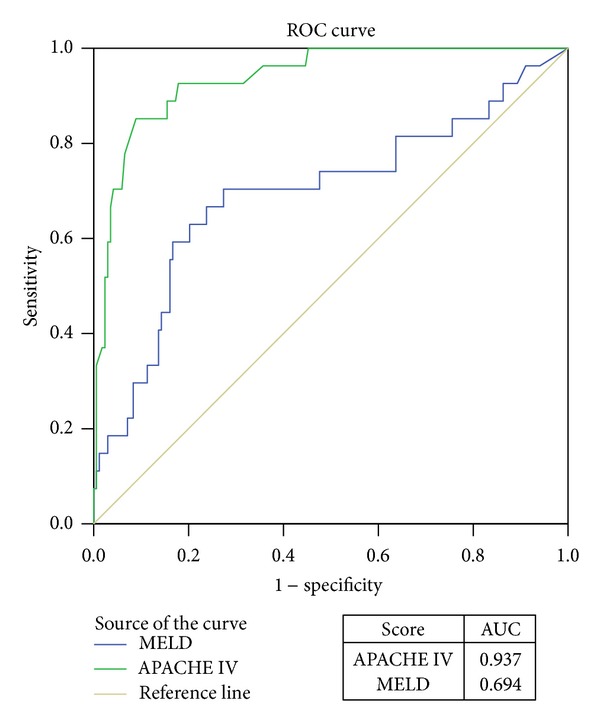
Receiver operating characteristic curves for the death risks of the acute physiology and chronic health evaluation IV (APAHCE IV) and the Model for End-stage Liver Disease (MELD) at preorthotopic liver transplantation (OLT). AUC, area under the receiver operating characteristic curve.

The AUC of the APACHE IV and MELD predictions of mortality during hospitalization was 0.937 (95%CI, 0.892 to 0.981) and 0.694 (95%CI, 0.51 to 0.817), respectively; *P* < 0.05 for both models. The two models were well-calibrated (with Hosmer-Lemeshow C statistics of 1.568 and 6.818 for APACHE IV and MELD, resp.; *P* > 0.05 for both models). The data was shown in Figure 1.

The highest Youden index was 0.763 when the APACHE IV score was 55.5 at the cutoff value, demonstrating a specificity of 0.911, a sensitivity of 0.852, a positive predictive value (PPV) of 0.605, and a negative predictive value (NPV) of 0.975. The highest Youden index was 0.430 when the MELD score was 20.7 at the cutoff value, demonstrating a specificity of 0.726, a sensitivity of 0.704, a positive predictive value (PPV) of 0.292, and a negative predictive value (NPV) of 0.938. In the combination test, the predicted mortality rate was classified as a positive result when APACHE IV score ≥ 55.5, and MELD score ≥ 20.07. The data was shown in Table 4.

The median predicted ICU LOS of survivors was 3.21 (2.39, 4.82) days, and the median actual ICU LOS was 3.71 (2.38, 5.47). There was a relationship between the predicted ICU LOS and the actual ICU LOS (*r* = 0.467, *P* < 0.05). Comparing the predicted ICU LOS to the actual ICU LOS of survivors, we noticed that the former was shorter than the latter (*Z* = −3.760, *P* < 0.05).

## 4. Discussion

The study put APACHE IV and MELD to evaluate their validity on posttransplantation of liver. We found that the AUC of APACHE IV was higher than that of MELD. We also demonstrated that nonsurvivors were higher in the mean APACHE IV score than survivors. Thus, our results may provide some guidance in the outcome judgment of patients after liver transplant.

Here, the hospital mortality was underestimated by using APACHE IV scoring system (SMR was 3.68, 95%CI: 2.38 to 4.96). In contrast to the original study by Zimmerman et al. [4],the SMR of 0.997 showed little difference between the predicted hospital mortality and the actual hospital mortality. Several reasons might account for this difference. First, our data might not be nationally representative because the collection was limited to patients who were admitted to the ICU after OLT. In addition, the recovery process of OLT patients was affected by the characteristics of the donors and the experience of the surgeons. At last, the different levels of ICUs may account for this discrepancy.

The ROC curves scoring system is used for predicting the sensitivity and specificity of death. The area under the receiver operating characteristic curve of APACHE IV was 0.937, however, higher than the value of 0.88 reported in the original study by Zimmerman et al. [4], suggesting that the APACHE IV score system has a good ability to distinguish possible nonsurvivors from survivors. This difference may be because the original data of Zimmerman for APACHE IV were derived mostly from integrated ICUs selected for complex diseases, while the subjects of this study were specific for ICU patients with OLT. The APACHE IV score was well-calibrated (Hosmer-Lemeshow was 1.568; *P* = 0.980). In contrast, other studies reported poorly calibrated APACHE IV scores that overestimated hospital mortality in integrated ICUs [7, 8]. According to our study, the APACHE IV score had a better calibration when it was applied to specialized ICUs, such as OLT patients; this scoring system is sensitive to distinguish possible nonsurvivors from survivors. Comparing with data from integrated ICUs, the APACHE IV score showed better predictive validity in a specialized ICU, which has been demonstrated by Knaus [9]. These results suggested that the APACHE IV scoring system is more appropriate for prediction of the prognosis of patients in specialized ICUs than in the integrated ICUs. 

The MELD score is based on 3 biochemical variables that are objective and easy to obtain, which are the international normalized ratio of prothrombin, serum creatinine, and serum bilirubin. Renal function is often recognized as a major determinant of patient survival and is given a heavy weight in MELD scoring system. In our study, the MELD score values in nonsurvivors (25.70 ± 12.92) were higher than in survivors (16.87 ± 9.61), *P* < 0.05. This result showed that the MELD score can predict early outcomes of transplantation and, as previously reported, hospital mortality. The pretransplantation MELD scores were 15~25, and the mortality rate was 6.12% at the lowest level; at <15, the mortality rate was 8.08%, and at >25, the mortality rate was 34.04%, which was the highest among the groups. The selection of proper patients and timing for OLT is complex and depends on multiple factors, such as survival, morbidity, resource utilization, and quality of life. Our results suggested that patients with lower death risks were not suitable for liver transplantation; in such cases, the survival times may be short. Therefore, the medium MELD scores (15~25) were the best fit for the operation; this confirms the results of Merion et al. that the low or high MELD score is not the most promising indicator [10].

The ROC curves scoring system was used to predict the sensitivity and specificity of death. The area under the receiver operating characteristic curve for MELD was 0.694, a relatively low prognostic value. The MELD scoring system was well-calibrated (Hosmer-Lemeshow was 6.818; *P* = 0.556). Basile-Filho et al. reported that the area under the receiver operating characteristic curve of MELD is only 0.5 [11]. The prognostic value to predict hospital mortality postoperation was low, which was also demonstrated in other studies [12, 13].

As there was no an objective and accurate evaluation tool available for prediction of the outcome for liver transplantation till now The highest Youden index was 0.430 when the MELD score was at a 20.07 cutoff value, demonstrating a specificity of 0.726, a sensitivity of 0.704, a positive predictive value (PPV) of 0.292, and a negative predictive value (NPV) of 0.938. This result indicated that APACHE IV was higher than MELD regarding sensitivity and specificity, which was mainly due to that APACHE IV takes the diagnosis at admission and objective data into account. Barie et al. reported that it would be better to combine APACHE IV with another critical scoring system to provide more accurate prediction [14]. In this study, although the combination of APACHE IV and MELD had the highest specificity and PPV, its Youden index was only 0.545. Vincent considered that different critical scoring systems may assist each other in their assessment, rather than compete with each other. The combined APACHE IV and MELD improved the predictive accuracy for postoperative mortality against MELD, but declined the accuracy when compared with APACHE IV. Thus, the Youden index was highest when using APACHE IV scores alone. Therefore, the function of the APACHE IV is better than that of others.

Prediction of ICU LOS by APACHE IV is used to evaluate and compare the overall efficient use of the ICU in medical center. Care in the ICU accounted for approximately 13% of hospital costs and 4.2% of national health expenditures [16]. These costs were largely explained by the LOS in the ICU [17, 18]. We found that the difference between the predicted ICU LOS and the actual ICU LOS was significant (*P* < 0.05), but the correlation between them was poor (*r* = 0.473, *P* < 0.05). The APACHE IV model provides clinically useful ICU LOS predictions for critically ill patient groups, but its accuracy and utility are still limited, as demonstrated in the study of Vasilevskis et al. [19]. 

In summary, the prognostic value of APACHE IV is higher than that of MELD scoring system; thus, it needs to be validated in multiple ICU centers. 

## Figures and Tables

**Table 1:** Comparison of survivors until hospital discharge and nonsurvivors (*n* = 195).

	All (195)	Survivors (*n* = 168)	Nonsurvivors (*n* = 27)	*P* values
Age, yrs, mean (S.D.)	48.18 (11.13)	47.83 (10.92)	50.41 (12.33)	0.265
Male	171	149	22	
Female	24	19	5	
MELD, mean (S.D.)	18.09 (10.55)	16.87 (9.61)	25.70 (12.92)	0.002
APACHE IV, mean (S.D.)	41.32 (21.95)	35.86 (15.58)	75.26 (25.47)	<0.001
Surgical time, hrs, mean (S.D.)	7.41 (1.61)	7.27 (1.58)	8.26 (1.60)	0.002
Anhepatic time, hrs, mean (S.D.)	42.30 (10.14)	41.76 (8.84)	45.59 (16.09)	0.370
Cold ischemia time, hrs, mean (S.D.)	7.13 (2.71)	7.11 (2.81)	7.23 (3.20)	0.688
Pred. hosp. death%, mean (S.D.)	3.76 (7.79)	2.30 (3.77)	12.84 (16.18)	<0.001
Pred.ICU LOS days, median (IQR)	3.49 (2.39, 4.82)	3.21 (2.21, 4.21)	5.5 (5.05, 6.03)	<0.001
Actual ICU LOS days, median (IQR)	3.71 (2.38, 5.47)	3.5 (2.34, 32.33)	8 (2.67, 32.75)	0.001
SMR; 95% CI	3.68; 2.38~4.96			

Hosp.: Hospital.

ICU: Intensive care unit.

Pred.: Predicted.

LOS: Length of stay.

Yrs: Years.

Hrs: Hours.

**Table 2:** Comparison of the mortality in APACHE IV score groups (*n* = 195).

	Group total	Nonsurvivors	APACHE IV	Actual mortality rates (%)	Actual mortality rates 95% CI	Pred. mortality rates (%)
All	195	27	41.32 ± 21.95	13.85	8.96~18.64	3.76
<30	65	0	21.77 ± 5.65			0.69
30~60	104	8	42.82 ± 8.18	7.69	2.57~12.81	2.62
>60	26	19	84.19 ± 21.62	73.08	52.00~88.00	16.00

**Table 3:** Comparison of the mortality in MELD score groups (*n* = 195).

Group	Total	Nonsurvivors	MELD score values	Actual mortality rate (%)
all	195	27	18.09 ± 10.55	13.85
<15	99	8	9.96 ± 2.72	8.08
15~25	49	3	19.10 ± 2.64	6.12
>25	47	16	34.02 ± 6.18	34.04

**Table 4:** APACHE IV, MELD, and combined APACHE IV and MELD score prognostic utility.

	Sensitivity	Specificity	PPV	NPV	Youden index	Cutoff value
APACHE IV	0.852	0.911	0.605	0.975	0.763	55.5
MELD	0.704	0.726	0.292	0.938	0.430	20.07
Combination	0.593	0.952	0.667	0.936	0.545	
